# Two pore domain THIK2 potassium channels regulate acute and chronic pain signaling

**DOI:** 10.1097/PR9.0000000000001481

**Published:** 2026-08-19

**Authors:** Nicolas Gilbert, Franck C. Chatelain, Solène Gibaud, Thomas Lorivel, Ying-Ling Shen, Marie-Emmanuelle Kerros, Sylvain Feliciangeli, Frederic Fiore, Chih-Cheng Chen, Florian Lesage, Delphine Bichet

**Affiliations:** a Université Côte d'Azur, CNRS, Inserm, Institut de Pharmacologie Moléculaire et Cellulaire (IPMC), Valbonne, France; b LabEx Ion Channel Science and Therapeutics (ICST), Valbonne, France; c Taiwan Mouse Clinic, National Comprehensive Mouse Phenotyping and Drug Testing Center, Academia Sinica, Taipei, Taiwan; d Centre d'Immunophénomique (CIPHE), Aix Marseille Université, Inserm, CNRS, CELPHEDIA, PHENOMIN, Marseille, France; e Institute of Biomedical Sciences, Academia Sinica, Taipei, Taiwan

**Keywords:** K2P channel, Excitability, Peripheral sensory neurons, Knock-out mice, Inflammatory pain

## Abstract

**Introduction:**

Two-pore domain potassium channels regulate neuronal excitability by generating background potassium currents that stabilize the resting membrane potential. Although several two-pore domain potassium channels have been implicated in pain signaling, the physiological role of tandem pore domain halotane-inhibited K+ channel (THIK2) remains largely unknown despite its high expression in human and mouse nociceptive dorsal root ganglion (DRG) neurons.

**Objective:**

This study aimed to determine the cellular distribution of THIK2 in sensory neurons and to investigate its contribution to neuronal excitability and nociceptive processing under physiological and inflammatory conditions.

**Methods:**

We combined molecular analyses, electrophysiological recordings, and behavioral approaches. THIK1 and THIK2 expression patterns were mapped in mouse DRGs. Whole-cell electrophysiology was used to assess membrane excitability in sensory neurons from wild-type and THIK2^−/−^ knockout mice. Behavioral tests evaluated thermal sensitivity under naive and inflammatory conditions.

**Results:**

We provide the first comprehensive characterization of THIK1 and THIK2 expression in mouse DRG. THIK2 deletion increased neuronal firing during sustained stimulation, indicating a loss of tonic inhibitory control of membrane excitability, particularly in nonpeptidergic IB4-positive C-fiber neurons. Behaviorally, THIK2^−/−^ mice displayed marked thermal hypersensitivity at baseline and during inflammation, consistent with enhanced sensory neuron hyperexcitability.

**Conclusion:**

THIK2 channels act as key regulators preventing pathological hyperexcitability in nociceptive sensory neurons. Their loss leads to increased neuronal firing and enhanced thermal sensitivity, identifying THIK2 as a promising therapeutic target for chronic inflammatory pain.

## Abstract 2

Supplemental Digital Content is Available in the Text.

A research study demonstrating that THIK2 channels, expressed in nonpeptidergic sensory neurons, exert an inhibitory control over neuronal excitability and reduce pain sensitivity in mice.

## 1. Introduction

Chronic pain has reached epidemic levels, and its treatment still relies heavily on nonsteroidal anti-inflammatory drugs and opioids,^32^ despite their numerous side effects.^37,45^ It is, therefore, essential to identify new pharmacological targets to develop safer analgesics. Ion channels have emerged as major drug targets because of their expression in sensory neurons and their central role in regulating excitability.^31^ In addition, several key regulators of sensory neuron function include receptor tyrosine kinases rearranged during transfection andtropomyosin receptor kinase, G protein-coupled receptors, and mechanotransduction-associated proteins (TACAN, stomatin-like protein 3, integrins).^6,9,23,65^ Pronociceptive channels, which depolarize neurons and favor action potential (AP) propagation, include sodium and calcium channels, transient receptor potential channels, acid-sensing ion channels, piezo channels, and purinergic P2X receptors. Several of them are key mediators of pain transduction, with transient receptor potential channels involved in chemical and thermal detection,^7,8,20,47,48^ acid-sensing ion channels in acidosis-induced pain,^22,25,35^ P2X receptors in inflammatory pain,^11,38,63,64^ and piezo channels in mechanical transduction.^9,29,41,53^ Recently, the peripheral sodium channel blocker suzetrigine demonstrated efficacy in phase III trials for moderate-to-severe acute pain.^54^ However, developing ion channel modulators remains challenging because of narrow therapeutic margins and side effects.

Potassium channels are promising antinociceptive targets because their activation inhibits APs and reduces pain signaling. Two-pore domain potassium (K2P) channels are particularly attractive because of their role in membrane hyperpolarization and their expression in pain pathways.^10^ They generate background potassium currents responsible for the negative resting membrane potential, acting as a brake on cellular excitability.^27,43^ In peripheral sensory neurons, this brake is essential for counteracting APs and preventing the transmission of nociceptive signals.^33^ Two-pore domain potassium channels include 15 genes (potassium channel subfamily K member) divided into 6 groups: two P-domain in a weakly inward rectifying K^+^ channel (TWIK), TWIK-related acid-sensitive K^+^ channel, TWIK-related alkaline-sensitive K^+^ channel, tandem pore domain halotane-inhibited K^+^ channel (THIK), TWIK-related K^+^ channel (TREK)/TWIK-related arachidonic acid activated K^+^ channel (TRAAK), and TWIK-related spinal cord K^+^ channel (TRESK).^28^ Several are expressed in trigeminal ganglia (TG) and dorsal root ganglia (DRG), where they modulate nociception. TREK1 channels colocalize with TRPV1 and substance P, while TREK1/TREK2 with IB4+ fibers,^1,68^ influencing neuronal excitability and spike firing.^2,52,55^ Behavioral studies from TREK/TRAAK knockout mice demonstrated altered sensitivity to mechanical and thermal stimuli in acute and chronic pain models.^4,17,57^ TRAAK activation by UV in rodents induces transient analgesia,^14^ and TREK1 channel agonists have shown analgesic effects but with potential side effects because of broad expression in the heart and central nervous system.^16,17,58^ TRESK channel is also widely expressed in sensory neurons from DRG and TG.^26,66^ Its inhibition selectively alters facial sensitivity under acute or pain-induced conditions, while producing little effect on general somatosensory responses elsewhere in the body.^3,19^ Finally, inactivation of TWIK-related acid-sensitive K^+^ channel 3 channels has been shown to decrease the cold pain threshold in living mice.^51^

Here, we examine the role of THIK2 in acute and chronic pain. THIK2 is the most highly expressed K2P channel in human DRGs and TGs,^12,30^ and in a subtype of C-fiber neurons of mouse DRGs.^12,69^ THIK2^−/−^ mice show increased thermal sensitivity under naive conditions and exhibit thermal/mechanical hyperalgesia under inflammatory conditions. We also show that THIK2 is mainly expressed in nonpeptidergic C-type DRG neurons, and describe, using electrophysiology, how its deletion alters neuronal excitability.

## 2. Methods

### 2.1. Animal strain and housing

Mouse strain, approvals, and housing conditions are detailed in the supplemental material (available at http://links.lww.com/PR9/A426).

### 2.2. Nociceptive behavioral assays

All behavioral experiments were performed on 20 to 25 g C57Bl/6J mice. Animals were acclimated for 45 minutes to their testing environment before any experiments. Experiments were made at room temperature (20–22°C) in a quiet room. All tests were conducted in a double-blind manner, without knowledge of the animals' genotype. The genotype was subsequently determined postmortem by genotyping a tail sample. von Frey and Hargreaves apparatus were from UgoBasile, Italy.

### 2.3. Formalin spontaneous pain test

Mice were placed individually into mirror chambers 45 minutes before injection, to allow them to acclimatize to their surroundings, and monitored with video. After intraplantar injection of 10 μL of a 2% formalin in NaCl 0.9% solution with a 31-gauge needle into the left hind paw, 4 nociceptive behaviors were quantified: time spent shaking, lifting, licking, or biting the injected paw was monitored for 45 minutes and analyzed in 5-minute intervals. This test can be separated in 2 phases: an initial acute phase I (0–10 minutes) followed by a relatively short quiescent period, and a prolonged tonic response (phase II from 10 to 45 minutes).^24,36^

### 2.4. Postformalin recovery test

A Noldus Phenotyper was used to analyze rearing and resting behavior. The phenotyper is equipped with a camera, and the videos were analyzed using Noldus Ethovision software. The animals were placed for 1 hour, without food or water, in a 30 × 30-cm box with clean bedding. This test was conducted a first time 10 days before the formalin test (naive condition), and a second time 1 hour after formalin 2% injection into the hind paw (post-formalin condition).

### 2.5. Mechanical sensitivity von Frey test

Mice were placed individually, 45 minutes before testing, into chambers with a wire mesh grid floor. Mechanical allodynia was either assessed using calibrated von Frey filaments ranging from 0.16 to 2 g or using dynamic von Frey apparatus with ramped increasing force (0–7.5 g) from Ugo Basile, Italy. For the manual test, the filaments tested in order of increasing stiffness, were applied 5 times, at more than 1-minute interval, perpendicularly to the plantar surface of the hind paw, and pressed until they bent. After the “ascending stimulus” method, the threshold was determined after 4 consecutive positive responses to the same filament.^24^ The thresholds obtained for each animal were then plotted as the percentage of responses at each threshold. For the dynamic von Frey test, a filament was applied 4 to 6 times to each hind paw using a force ramp. The force required to elicit paw withdrawal (threshold) was recorded, and the means of these thresholds were calculated for each animal.

### 2.6. Radiant heat Hargreaves test

Mice were placed individually, 45 minutes before testing, into chambers with a plexiglass floor. Thermal radiant heat was assessed using the Hargreaves device calibrated at 50% and 60% of power. Approximately 50% power (260 mW/cm^2^) was used to produce withdrawal latencies of 10 to 12 seconds in naive animals.^34^ The threshold was determined by averaging 4-8 values for each hind paw of each animal.

### 2.7. Complete Freund adjuvant–induced inflammatory hypersensitivity

We made an intraplantar injection of 15 μL of a 1:1 complete Freund adjuvant (CFA) (ThermoScientific, France) in saline emulsion with a 26-gauge needle. We then measured the thermal and mechanical thresholds as described above every 2 or 4 days until day 28 post-CFA injection. This method is a well-established approach to model chronic inflammatory pain.^18,50^

### 2.8. X-gal dorsal root ganglion staining

X-gal staining was performed as described in the supplemental material (available at http://links.lww.com/PR9/A426).

### 2.9. Fluorescent in situ hybridization

In situ hybridization and immunofluorescence were conducted following RNAscope Multiplex V2 protocol for fresh frozen tissues from Bio-techne. To obtain adult tissues, mice were anesthetized with isoflurane (Vetflurane) and decapitated. Then, L1 to L6 DRG were dissected, immediately placed in cryoprotective optimal cutting temperature solution before being frozen, and stored at −80°C. Dorsal root ganglia were sectioned at 10 μm (DRG section) using a Leica CM3050S cryostat. Specific probes were used to detect *Kcnk12* (THIK2), *Kcnk13* (THIK1), *P2rx3* (purinergic receptor X 3), *Calca* (CGRP, calcitonin gene–related peptide), *Th* (tyrosine hydroxylase), and *Nefh* (NF200, neurofilament 200) mRNAs. Hybridization with a probe against the *Bacillus subtilis* *dapB* (dihydrodipicolinate reductase) gene (Ref. 320871) was used as a negative control, and a mix of *Ppib* (peptidylprolyl isomerase B), *Ubc* (ubiquitin C), and *Pol2ra* (RNA polymerase II subunit A) (Ref. 320881) was used as a positive control. Probes were hybridized 2 hours at 40°C. Final detection was achieved using TSA Vivid kit 520, 570, and 650 (TOCRIS bio-techne, France). Z-stack images were acquired using a laser-scanning confocal microscope (TCS SP8 Leica microsystem, France) with a 63X objective. Quantifications were done using QuPath v0.5.0 software, with area automatically determined by the software after identification of the labelled neurons. The number of dots per cell was detected after applying the same detection threshold to all images. For each genotype, lumbar DRG neurons were counted in 3 independent animals.

### 2.10. Dorsal root ganglion neuron culture and patch-clamp recordings

Dorsal root ganglion neurons were obtained from 8- to 12-week-old wild type (WT; C57Bl/6J) and THIK2^−/−^ mice. After isoflurane anesthesia, animals were decapitated, lumbar DRG were retrieved, digested enzymatically with collagenase, and plated in 35-mm petri dishes coated with poly-(l)-lysine and cultivated in Neurobasal. Patch-clamp experiments were performed 1 to 3 days after plating. Nonpeptidergic C-fiber neurons were determined by Isolectin B4 staining using IB4 (1:2000) conjugated with AlexaFluorR 488, applied 4 hours before recordings. All recordings were made at room temperature (20–22°C). External solution contained (in mM): 140 sodium chloride, 4 potassium chloride, 1 calcium chloride, 1 magnesium chloride, 10 4-(2-hydroxyethyl)-1-piperazineethanesulfonic acid (pH 7.4 adjusted with sodium hydroxide). Patch pipettes had a resistance of 3.5 to 5.5 MΩ and were filled with (in mM) 155 potassium chloride, 4 magnesium adenosine triphosphate, 5 ethylene glycol-bis(β-aminoethyl ether)-N,N,N′,N′-tetraacetic acid and 10 4-(2-hydroxyethyl)-1-piperazineethanesulfonic acid (pH 7.2 with potassium hydroxide). Voltage signals were recorded using a 700B Multiclamp amplifier (Axon Instruments), low-pass Bessel-filtered at 1 kHz, and digitized at 10 kHz by using a Digidata-1550B (Axon Instrument). Clampex was used for current visualization and stimulation. Current-clamp: membrane potential was recorded under control bath solution perfusion. Rheobase was obtained after successive application of 20 pA until an AP was obtained. The protocol consisted of applying 10 sweeps, each sweep consisting of stimulation with a current equal to 3× the rheobase for 5 milliseconds to trigger an AP. After 500 milliseconds of rest, we applied again a current equal to 3× the rheobase for 800 milliseconds to trigger trains of APs, followed by 1 second of rest. Data were obtained and analyzed with Clampfit software. The different parameters were determined and analyzed as described in Sun et al.^61^

### 2.11. Statistical analysis

All statistical tests are performed using GraphPad Prism 10.4.1 software and described in the figure legends.

## 3. Results

### 3.1. THIK1 and THIK2 are selectively co-expressed in non-peptidergic dorsal root ganglion neurons

**Figure 1.**
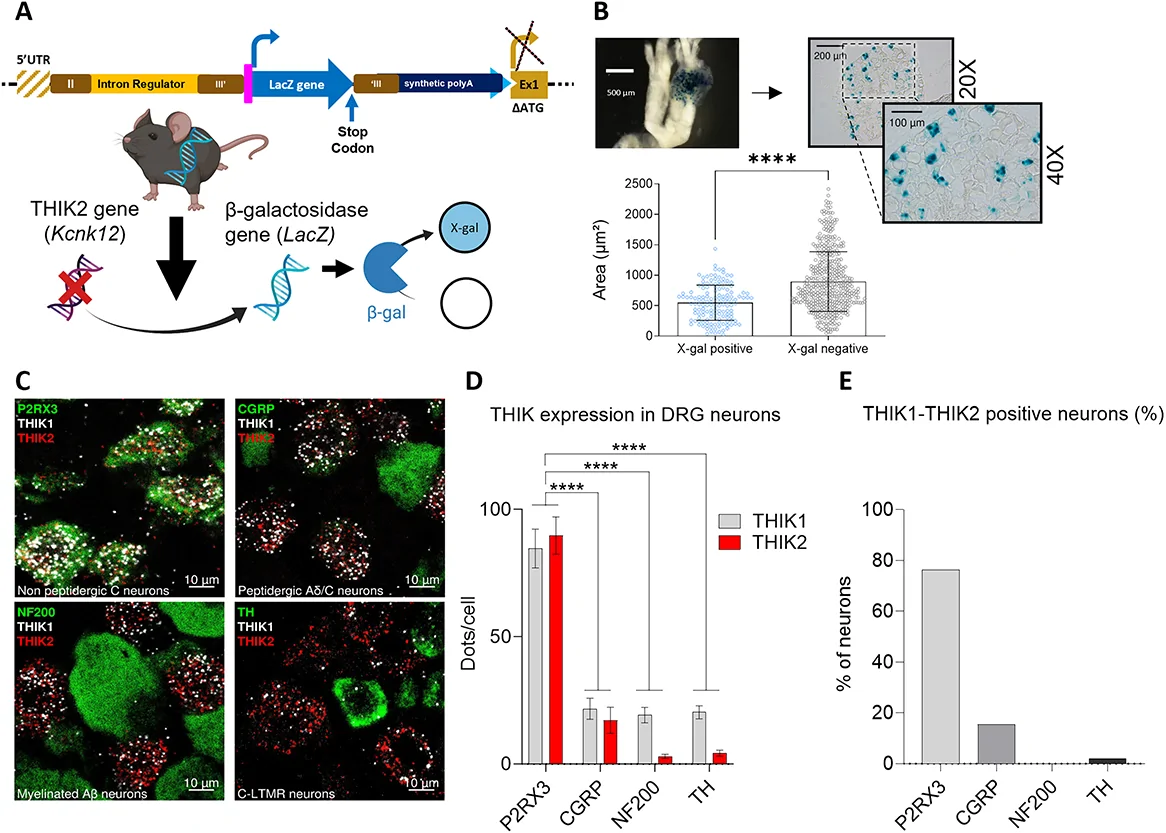
THIK2 expression mapping in DRG neurons based on combined X-gal staining and RNAscope analysis showing selective expression in small-diameter neurons and colocalization with P2RX3-positive non-peptidergic nociceptors. (A) The THIK2^−/−^βGal^+/+^ mouse was made by inactivation of the *kcnk12* gene and introduction of the *LacZ* gene as described in supplemental materials (available at http://links.lww.com/PR9/A426). The *LacZ* gene codes for β-galactosidase (β-gal) in THIK2^−/−^βGal^+/+^ mice, which turns X-gal compound in blue precipitate. Created with BioRender.com. (B) Images of β-gal labeled whole lumbar DRG from 5 different THIK2^−/−^βGal^+/+^ homozygous male mice. DRG cryostat sections are 10-μm thick, and magnifications used are ×20 and ×40. Quantification of β-gal positive (n = 143) and negative (n = 453) cells. Mean ± SEM, unpaired *T* test, ****P < 0.0001. (C) Confocal images of mouse lumbar DRG sections colabeled with mRNA probes for THIK2 (red), THIK1 (white) and neuronal markers for nonpeptidergic C neurons (P2RX3), peptidergic Aδ/C neurons (CGRP), myelinated Aβ large neurons (NF200), and C-low threshold mechanoreceptor neurons, C-LTMR (TH). (D) Quantification of THIK2 and THIK1 mRNAs dots in P2RX3 (n = 76), CGRP (n = 71), NF200 (n = 80), and TH (n = 48) positive DRG neurons from 3 independent male mice. Mean ± SEM, 2-way ANOVA with Tukey multiple comparisons test, ****P < 0.0001. (E) Neurons positivity (>20 dots per cell) percentage for coexpression of THIK1 and THIK2 mRNAs along with each neuronal marker. ANOVA, analysis of variance; CGRP, calcitonin gene–related peptide; DRG, dorsal root ganglion; NF200, neurofilament 200; P2RX3, purinergic receptor X 3; THIK, tandem pore domain halotane-inhibited K^+^ channel.

THIK1 and THIK2 are selectively coexpressed in non-peptidergic DRG neurons. To study THIK2 expression in peripheral sensory systems, we created reporter mice carrying β-galactosidase in place of the *Kcnk12* gene coding for THIK2 subunit (Fig. 1A). X-gal staining on lumbar DRGs from homozygous THIK2^−/−^ βGal^+/+^ mice revealed clear labeling appearing as dot-like patterns at ganglion level, resembling sensory neuron cell body labeling (Fig. 1B). Detailed analysis of 10 μm DRG sections showed X-gal staining in subsets of structures. Size distribution analysis showed higher X-gal staining proportion in small and medium-sized neurons compared with X-gal negative neurons (Fig. 1B), with averaged size of X-gal positive neurons being 549.5 ± 2.0 μm^2^, which correlates with the area of small C-fiber neurons (<650 μm^2^)^65^ compared with 897.1 ± 1.1 μm^2^ for X-gal negative neurons.

Dorsal root ganglion neurons were molecularly characterized using RNAscope fluorescent in situ hybridization to examine THIK2 with markers including NF200, P2RX3, CGRP, and TH (Fig. 1C). Our findings revealed that THIK2 is predominantly expressed in P2RX3-positive neurons (Fig. 1D), corresponding to non-peptidergic C-fiber nociceptors, in line with RNA-seq data showing that THIK2 transcripts are the most abundant K2P transcripts in this neuronal population.^69^ Quantification revealed that P2RX3 neurons (89.72 ± 7.34 dots per cell) exhibited THIK2 levels approximately 5 times higher than in CGRP neurons (17.16 ± 5.12 dots per cell), with minimal detectable THIK2 labeling in TH (4.29 ± 1.17 dots per cell) and NF200 (3.03 ± 0.82 dots per cell) neurons (Fig. 1D).

RNAscope experiments revealed that both THIK1 and THIK2 are coexpressed in P2RX3-positive neurons, accounting for more than 76% of cells (Fig. 1E). THIK1 displays an expression pattern similar to THIK2, indicating potential homodimer and/or heterodimer formation between channels in these neurons, as we know that they can form functional heteromers.^15,40^ Analysis showed no increase in THIK1 expression patterns following THIK2 deletion; instead, we observed a statistically significant decrease in THIK1 levels in P2RX3, CGRP, and NF200 neurons, corroborated by qPCR analysis extending to other K2P family channels and pain-implicated channels (Supplementary Fig. S1, available at http://links.lww.com/PR9/A426).

### 3.2. Increased excitability of non-peptidergic dorsal root ganglion neurons from THIK2^−/−^ mice

**Figure 2.**
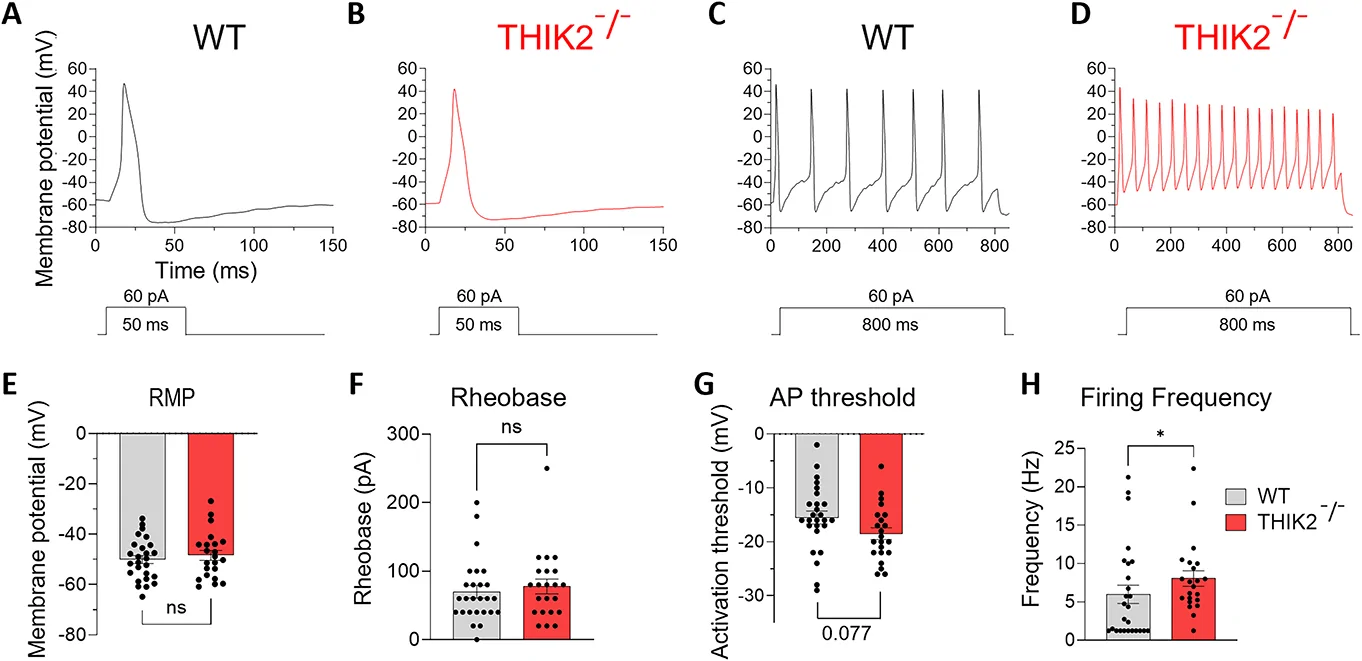
Patch-clamp recordings from IB4-positive neurons showing hyperexcitability of THIK2^−/−^ neurons. (A and B) Examples of AP evoked by a single 50-millisecond depolarizing current pulse at 60 pA (3× rheobase) in IB4-positive neuron from WT (black) and THIK2^−/−^ (red) mice. The injected currents range from a minimum of 60 pA up to 750 pA, depending on the rheobase determined for each neuron. The stimulation protocol is shown below the corresponding graph. (C and D) Examples of AP trains evoked by a single 800-millisecond depolarizing current pulse at 60 pA (3 × rheobase) in an IB4-positive neuron from WT (black) and THIK2^−/−^ (red) mice. The stimulation protocol is shown below the corresponding graph. (E) Average RMP values in IB4-positive neurons from WT (grey) and THIK2^−/−^ (red) mice. (F) Average rheobase, minimum current to elicit AP, in IB4-positive neurons from WT (grey) and THIK2^−/−^ (red) mice. (G) Average AP threshold potential of IBA4-positive neurons from WT and THIK2^−/−^ mice. (H) Average firing frequency calculated as the number of APs generated in response to a single 800-millisecond stimulation at 3× rheobase (seen in Fig. 2C and D) divided by the stimulation time in IB4-positive neurons from WT (grey) and THIK2^−/−^ (red) mice. Parameters were determined on nonpeptidergic IB4-positive from WT (n = 26) or THIK2^−/−^ (n = 22) neurons. Mean ± SEM, unpaired *T* test (F–G) and Mann–Whitney test (E–H), *P < 0.05. AP, action potential; RMP, resting membrane potential; THIK, tandem pore domain halotane-inhibited K^+^ channel; WT, wild-type.

Electrophysiological studies on cultured DRG neurons from THIK2^−/−^ mice were conducted on small- to medium-sized neurons and IB4-positive neurons to target nonpeptidergic C-nociceptors (Fig. 2A–D). Membrane capacitance measurements indicated no significant difference between WT and THIK2^−/−^ neurons (39.9 ± 0.6 pF for WT vs 41.6 ± 0.5 pF for THIK2^−/−^), suggesting comparable cell sizes (Supplementary table, available at http://links.lww.com/PR9/A426). Resting membrane potential was slightly more depolarized in THIK2^−/−^ neurons (−48.4 ± 0.4 mV) compared with WT (−50.1 ± 0.3 mV), though not statistically significant (mean ± SEM; Fig. 2E).

Rheobase determination showed no significant difference for THIK2^−/−^ neurons (77.7 ± 2.3 pA) compared with WT (70 ± 1.8 pA) (Fig. 2F). Action potential threshold was also slightly different (−18.5 ± 0.2 mV for THIK2^−/−^ vs −15.5 ± 0.2 mV for WT; Fig. 2G). However, during prolonged stimulation (800 milliseconds), THIK2^−/−^ neurons exhibited significantly higher firing frequency (8 ± 0.2 Hz) compared to WT neurons (6 ± 0.2 Hz, *P* = 0.047; Fig. 2H). Notably, 95.45% of THIK2^−/−^ neurons displayed at least 2 AP responses, whereas only 57.69% of WT neurons did so. Action potential parameters, assessed using stimulation at 3 × rheobase, including peak potential and AP duration, showed minimal or no differences between WT and THIK2^−/−^ neurons (Supplementary table, available at http://links.lww.com/PR9/A426). These findings suggest that THIK2 current plays a limited role in most aspects of AP generation but rather influences AP frequency.

### 3.3. Acute mechanical and thermal sensitivity in THIK2^−/−^ mice

**Figure 3.**
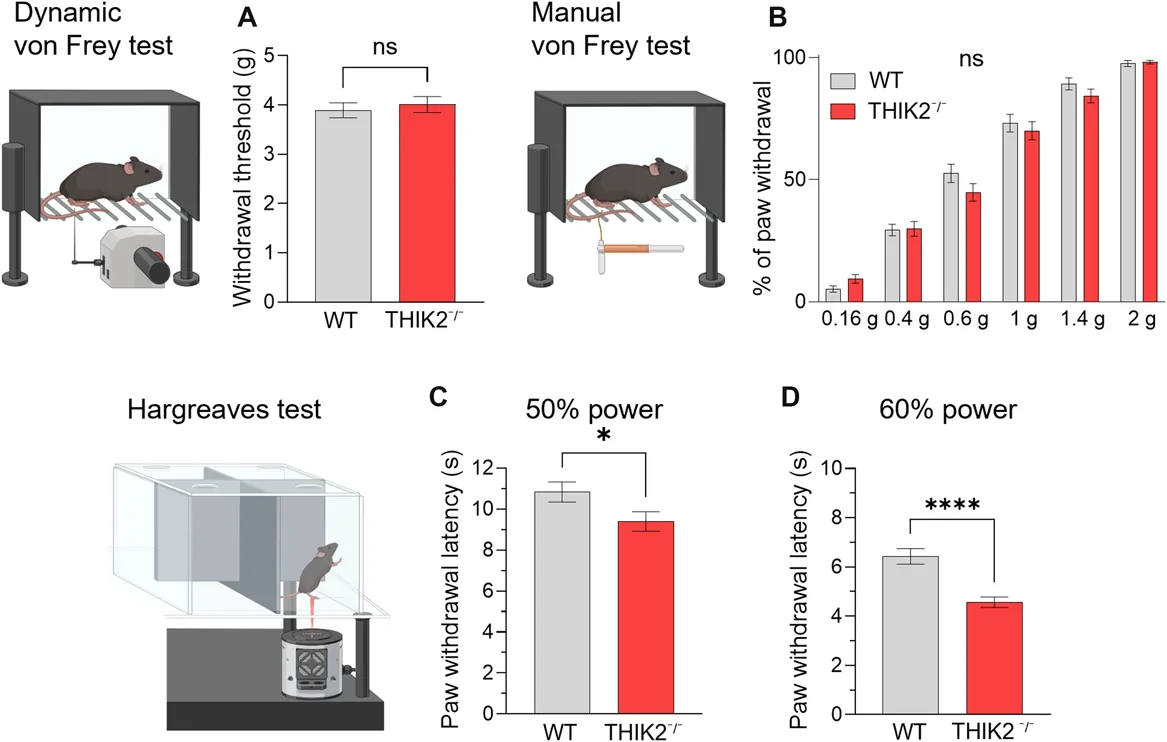
Thermal hypersensitivity of THIK2^−/−^ mice. (A) Paw withdrawal threshold (g) of THIK2^−/−^ (n = 19) compared to WT (n = 20) mice in response to mechanical stimuli using the dynamic von Frey test (1–7.5 g ramp). Data are presented as mean ± SEM; unpaired *T* test. (B) Percentage of paw withdrawals of THIK2^−/−^ (n = 48) compared to WT (n = 48) mice in response to mechanical stimuli using the manual von Frey test (0.16–2 g). Data are presented as mean ± SEM; 2-way ANOVA. (C) Paw withdrawal latency (s) of THIK2^−/−^ (n = 20) compared with WT (n = 18) mice in response to a 50% thermal stimuli intensity in the Hargreaves test. Data are presented as mean ± SEM; unpaired *T* test, *P < 0.05. (D) Paw withdrawal latency (s) of THIK2^−/−^ (n = 46) compared to WT (n = 48) mice in response to a 60% thermal stimuli intensity in the Hargreaves test. Data are presented as mean ± SEM; unpaired *T* test, ****P < 0.001. Illustrations were created with BioRender. ANOVA, analysis of variance; THIK, tandem pore domain halotane-inhibited K^+^ channel; WT, wild-type.

Acute mechanical and thermal sensitivities were assessed using the von Frey and Hargreaves tests, by applying calibrated filaments (manual von Frey test) or a continuous force ramp (dynamic von Frey test) and radiant heat (Hargreaves test) to the hind paw and measuring paw withdrawal thresholds or latencies. Behavioral assessment revealed no differences in mechanical sensitivity between THIK2^−/−^ and WT mice (Fig. 3A and B). Hargreaves testing at 50% (260 mW/cm^2^) power revealed thermal allodynia for THIK2^−/−^ mice (Fig. 3C). Paw withdrawal thresholds were 10.84 ± 0.49 seconds for WT compared with 9.39 ± 0.48 seconds for THIK2^−/−^ mice, showing nearly 15% decrease (Fig. 3C), with gender-related differences where thermal hypersensitivity was exclusively observed in females at this intensity (Supplementary Fig. S2, available at http://links.lww.com/PR9/A426). At 60% power applied to larger cohorts, thermal hypersensitivity was observed in both sexes (Fig. 3D; Supplementary Fig. S2, available at http://links.lww.com/PR9/A426). Withdrawal thresholds were 6.43 ± 0.32 seconds for WT compared to 4.57 ± 0.21 seconds for THIK2^−/−^ mice, showing almost 30% decrease (Fig. 3D). These data underscore the importance of THIK2 channels for heat sensitivity and represent the first demonstration of a THIK channel role in nociception.

### 3.4. Enhanced inflammatory acute and chronic pain responses in THIK2^−/−^ mice

**Figure 4.**
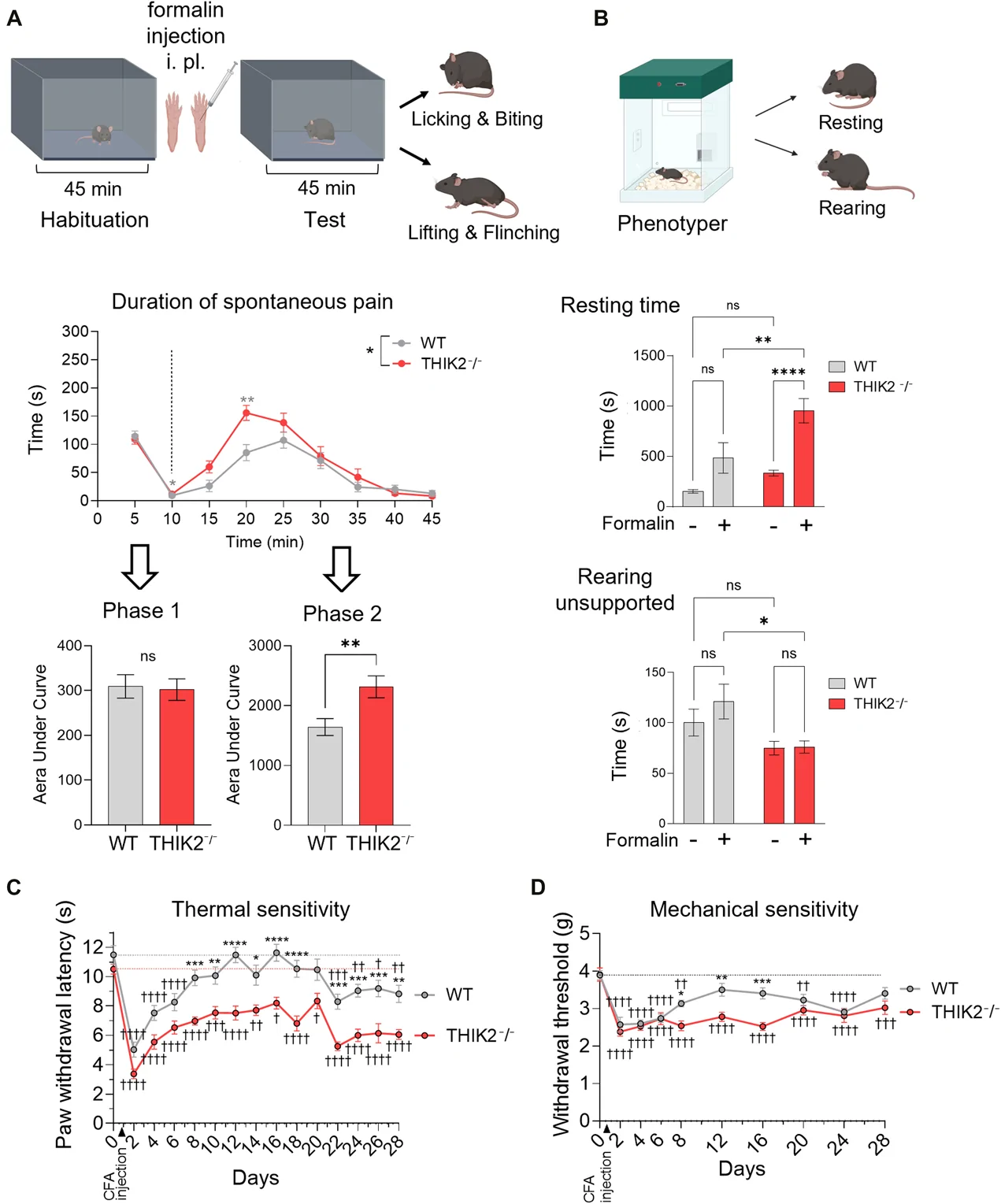
THIK2^−/−^ mice exhibit enhanced and sustained inflammatory pain under acute and chronic inflammatory conditions. (A) Duration of spontaneous pain behaviors in WT (n = 20) and THIK2^−/−^ (n = 24) mice during the formalin-induced pain test including licking, biting, shaking, and paw lifting. The test lasted 45 minutes after intraplantar (i.pl.) injection of 15 μL of formalin into the left hind paw. Data are presented at mean ± SEM; 2-way ANOVA with Tukey multiple comparisons test, *P < 0.05, **P < 0.01. AUC of pain behaviors was analyzed on 2 phases: phase 1 (acute chemical phase, 0–10 minutes) and phase 2 (acute inflammatory phase, 10–45 minutes). Data are presented at mean ± SEM; unpaired *t* test, **P < 0.01. (B) Postformalin recovery was assessed in WT (n = 18) and THIK2^−/−^ (n = 23) mice by comparing behaviors before formalin injection (−) and 15 minutes after completion of the formalin test (+). Animals were individually placed in a Phenotyper observation cage for 1 hour, during which resting/prostration time and time spent rearing were quantified. Data are presented at mean ± SEM; 2-way ANOVA with Tukey multiple comparisons test, *P < 0.05, **P < 0.01, and ****P < 0.001. (C) Paw withdrawal latency (s) measured in response to thermal stimuli (Hargreaves test, 50% power) before and during 28 days after CFA injection into the hind paw of WT (n = 20) and THIK2^−/−^ mice (n = 19). (D) Paw withdrawal force threshold (g) measured in response to mechanical stimuli (dynamic von Frey test, 1–7.5 g ramp) before and during 28 days after CFA injection into the hind paw of WT (n = 20) and THIK2^−/−^ mice (n = 19). For panels C and D, data are presented as mean ± SEM. Statistical analyses were performed using 2-way ANOVA with Bonferroni multiple comparisons test for comparisons with THIK2^−/−^ (*P < 0.05, **P < 0.01, ***P < 0.005, ****P < 0.001) and Dunnett multiple comparisons test for comparisons with day 0 (D0) (†*P* < 0.05, ††*P* < 0.01, †††*P* < 0.005, ††††*P* < 0.001). Illustrations were created with BioRender.com. ANOVA, analysis of variance; AUC, area under curve; CFA, complete Freund adjuvant; THIK, tandem pore domain halotane-inhibited K^+^ channel; WT, wild-type.

The formalin plantar spontaneous pain test was used to assess acute chemical and inflammatory pain (Fig. 4A). A 2% formalin solution was injected into the hind paw, and spontaneous pain behaviors were recorded over a 45-minute period. No significant difference was observed during acute chemical pain phase (0–10 minutes) between THIK2^−/−^ and WT mice (Fig. 4A). However, during the acute inflammatory phase (10–45 minutes), THIK2^−/−^ mice exhibited a significant increase in spontaneous pain behaviors, such as licking, biting, lifting and flinching of the hind paws, with a peak observed at 20 minutes (Fig. 4A). Area under curve from acute inflammatory phase 2 was significantly 40% higher for THIK2^−/−^ mice (2314.03 ± 183.49) compared to WT mice (1643.37 ± 140.87), confirming inflammatory hyperalgesia phenotype (Fig. 4A). Male and female mice, as well as WT littermates from the same lineage as THIK2^−/−^ mice, were tested, with no differences observed between sexes or between WT from Janvier Labs and WT littermates (Supplementary Fig. S3, available at http://links.lww.com/PR9/A426).

To assess potential discomfort-related behaviors during the postinflammatory phase, mice were placed in a videotracked observation cage (PhenoTyper) to monitor spontaneous activity (Fig. 4B). Resting (immobility) and rearing (time spent standing on the hind paws) behaviors were quantified to evaluate whether mice exhibited signs of persistent discomfort. Each animal was tested twice for 1 hour: first in a naive condition prior to the formalin test and a second time after the formalin test, when paw inflammation was present but spontaneous pain behaviors had resolved. Postformalin recovery assessment revealed that THIK2^−/−^ mice exhibited a 2-fold increase in resting time (952.94 ± 120.96 seconds) compared with WT mice (484.52 ± 151.10 seconds; Fig. 4B), together with a significant reduction in rearing behavior (75.95 ± 6.06 seconds vs 121.10 ± 17.29 seconds in WT; Fig. 4B). The prolonged resting time reflects a discomfort-like state, as animals remain immobile with no sign of exploratory movement. Similarly, the reduced rearing behavior indicates a persistent discomfort or reluctance to use the affected limb. Sex differences were observed, with female THIK2^−/−^ mice exhibiting longer resting times, reflecting reduced recovery compared with their male counterparts (Supplementary Fig. S3, available at http://links.lww.com/PR9/A426).

In the chronic inflammatory hypersensitivity test, baseline mechanical and thermal withdrawal thresholds were first measured using von Frey and Hargreaves tests (Fig. 4C and D). Mice then received an intraplantar injection of CFA into the hind paw to induce strong peripheral inflammation, and the same behavioral assessments were performed over the following month to monitor the development and persistence of hyperalgesia. Complete Freund adjuvant–induced chronic inflammatory hypersensitivity over 28 days revealed that THIK2^−/−^ mice exhibited sustained thermal hyperalgesia throughout the testing period, with a significant difference between THIK2^−/−^ and WT mice emerging by day 8 and lasting until day 28 (Fig. 4C). In mechanical von Frey testing, THIK2^−/−^ mice also displayed sustained mechanical hyperalgesia over the 28-day period, with significantly greater sensitivity compared with WT mice between days 8 and 18 (Fig. 4D). Although WT mice progressively recovered to baseline by day 28, THIK2^−/−^ mice failed to recover (Fig. 4D). In both mechanical and thermal CFA-induced chronic inflammatory tests, occasional sex-related differences were observed in both WT and THIK2^−/−^ mice, without affecting the overall hyperalgesic phenotype of THIK2^−/−^ mice (Supplementary Fig. S4, available at http://links.lww.com/PR9/A426).

No other phenotypes related to mechanical and chemical itch or anxiety and locomotion were observed in THIK2^−/−^ mice (Supplementary Figs. S5 and S6, available at http://links.lww.com/PR9/A426).

## 4. Discussion

Overall, our results demonstrate that THIK2 acts as an inhibitor of nociceptive transmission by reducing excitability. THIK2 is selectively expressed in nonpeptidergic C-fiber neurons (Fig. 1), which are well known for their role in nociceptive detection and transmission during inflammatory pain.^56^ Electrophysiological recordings show that THIK2 modulates membrane excitability in sensory neurons, with an increased number of APs in THIK2^−/−^ neurons during sustained stimulation (Fig. 2). This result highlights a tonic regulatory role of THIK2, likely acting as a brake that prevents pathological hyperexcitability in non-peptidergic, IB4-positive C-fiber neurons. These electrophysiological data are consistent with the hyperalgesia seen in THIK2^−/−^ animals (Figs. 3 and 4), suggesting that THIK2 normally contributes to decreasing C-fiber nociceptors firing.

THIK2^−/−^ mice exhibit basal thermal hyperalgesia in the absence of inflammatory stimuli, along with heightened spontaneous pain in chemically induced inflammation models. In naive conditions, the absence of a mechanical phenotype in the von Frey test likely reflects both redundancy among mechanosensitive afferents and the low-intensity nature of the stimulus, whereas the Hargreaves test, which delivers a sustained and clearly noxious thermal stimulus, more effectively reveals the impact of THIK2 loss on the excitability of polymodal nonpeptidergic fibers. During chronic inflammation, these mice display marked mechanical and thermal hyperalgesia, confirming the key role for THIK2 in polymodal nociceptors, particularly nonpeptidergic C-fiber neurons that transmit slow-conducting pain signals and respond to diverse stimuli in inflammatory states.^39,42^ Evidence for the selective role of THIK2 comes from the observation that mechanical hyperalgesia in THIK2^−/−^ mice only appears after 8 days of inflammation. Given that peripheral sensitization typically begins around day 2, when normally Aβ fibers start to convey pain signals,^60^ the delayed onset of mechanical hyperalgesia suggests that it results from increased excitability of slow conducting polymodal C fibers rather than sensitization of Aβ fibers. The delayed onset of mechanical hypersensitivity in THIK2^−/−^ mice may also suggest that THIK2 is upregulated during inflammation to limit neuronal excitability, with its absence unmasking the phenotype; this will require further investigation of THIK and other K2P channel expression. These mice are global knockouts and not DRG-specific, which does not allow us to exclude a potential additional role of THIK2 in other structures that may, or may not, contribute to the pain phenotype. Notably, THIK2 expression has also been detected in several regions of the central nervous system. Future studies using conditional gene inactivation will be essential to analyze the respective contributions of peripheral and central THIK2 in pain processing.

If THIK2 acts as a brake on sensory excitability, its activation by selective agonists should, therefore, provide analgesia. The advantages of targeting THIK2 rather than other channels for pain modulation become apparent when considering the broader roles of the K2P channels in pain pathophysiology. Genetic ablation of the K2P TRESK channel enhances mechanical and cold sensitivity while leaving heat sensitivity largely unchanged.^3,19^ TRESK is preferentially expressed in NP1 and NP2 neuron subtypes involved in itch signal transmission. Recent studies show that TRESK deletion exacerbates chronic itching in several mouse models.^44^ TRAAK is ubiquitously expressed in all types of sensory fibers, and its genetic ablation results in increased thermal and mechanical pain.^52^ TREK1 and TREK2 are both present in C-fiber nociceptors, with TREK1 preferentially localized in peptidergic neurons.^49^ These 2 channels contribute significantly to the electrical stability of DRG neurons, as their blockade increases neuronal excitability and causes hyperalgesia. Several small-molecule activators of TREK and TRAAK channels have been evaluated for their analgesic potential, but most candidates exhibit unwanted systemic effects including hypotension, sedation, and muscle weakness.^16,62^

Recent transcriptomic and proteomic studies confirmed the expression of THIK2 in human DRG, providing an essential translational link. THIK2 is expressed in the same nociceptor subtypes in humans as in mice, ranking among the 10 most abundantly expressed ion channels in human DRG neurons.^5,12^ This conserved expression profile reinforces the relevance of our findings and highlights THIK2 as a potential key candidate in the pathophysiology of human pain. RNAscope analyses demonstrated strong coexpression of THIK1 and THIK2 in P2RX3-positive neurons, suggesting potential formation of heterodimers. The slight downregulation of THIK1 mRNA in THIK2^−/−^ mice indicates no compensatory mechanism, reinforcing the validity of the knockout model for studying the specific roles of THIK2. THIK1 has been implicated in microglial surveillance and NLRP3 inflammasome activation in the central nervous system, where inhibition rather than activation of THIK1 is envisaged to reduce inflammation.^46,59^ THIK2 channels have been reported to localize predominantly intracellularly under heterologous expression conditions.^13,21^ Covalent binding experiments with THIK1 suggest that THIK1 may facilitate THIK2 trafficking to the plasma membrane.^15^ Although it remains unknown whether THIK2 requires THIK1 to reach the neuronal membrane in sensory neurons, we show that the absence of THIK2 affects neuronal excitability even when THIK1 is present, indicating that THIK1 alone is insufficient to compensate for its loss. In contrast, THIK2 homomers may regulate excitability either directly by setting the membrane potential or indirectly through modulation of intracellular calcium homeostasis. Given the minimal differences in intrinsic biophysical properties between THIK2 homomers and THIK1/THIK2 heteromers, distinguishing their respective contributions remains challenging in the absence of a heteromer-specific modulator. In this context, a combined THIK1/THIK2 knockout could help clarify the relative roles of THIK2 homomers vs THIK2-containing heteromers.

Overall, our results reveal a novel and specific role for THIK2 in thermal and inflammatory nociception regulation, particularly via nonpeptidergic C-fiber neurons. The minimal compensatory changes observed in the expression of other ion channels in THIK2^−/−^ mice confirm the non-redundant function of THIK2. Together with its conserved expression profile in human sensory neurons, this highlights THIK2 as a promising therapeutic target, especially in the context of chronic inflammatory pain and opioid-sparing strategies. However, the development of selective THIK2 modulators, particularly activators,will be essential, as no such compounds have been reported to date, either as activators or inhibitors. As an important intermediate step, demonstrating that THIK2 activation, for instance through a gain-of-function mutation, can produce beneficial analgesic effects in vivo with minimal side effects would further support the therapeutic relevance of targeting this channel. Future studies will, therefore, aim to identify and validate selective THIK2 modulators in various preclinical pain models and assess their safety and efficacy in clinically relevant conditions.

## Disclosures

The authors have no conflicts of interest to declare.

## Supplemental digital content

Supplemental digital content associated with this article can be found online at http://links.lww.com/PR9/A426.
