# Supplementary material for: Two pore domain THIK2 potassium channels regulate acute and chronic pain signaling
Source: Pain Rep. 2026 Aug 19;11(5):e1481. doi: 10.1097/PR9.0000000000001481 (PMC13492956; doi:10.1097/PR9.0000000000001481)
Supplement: Supplementary file 1 [file painreports-11-e1481-s001.pdf]

SUPPLEMENTAL DIGITAL CONTENT

Figures

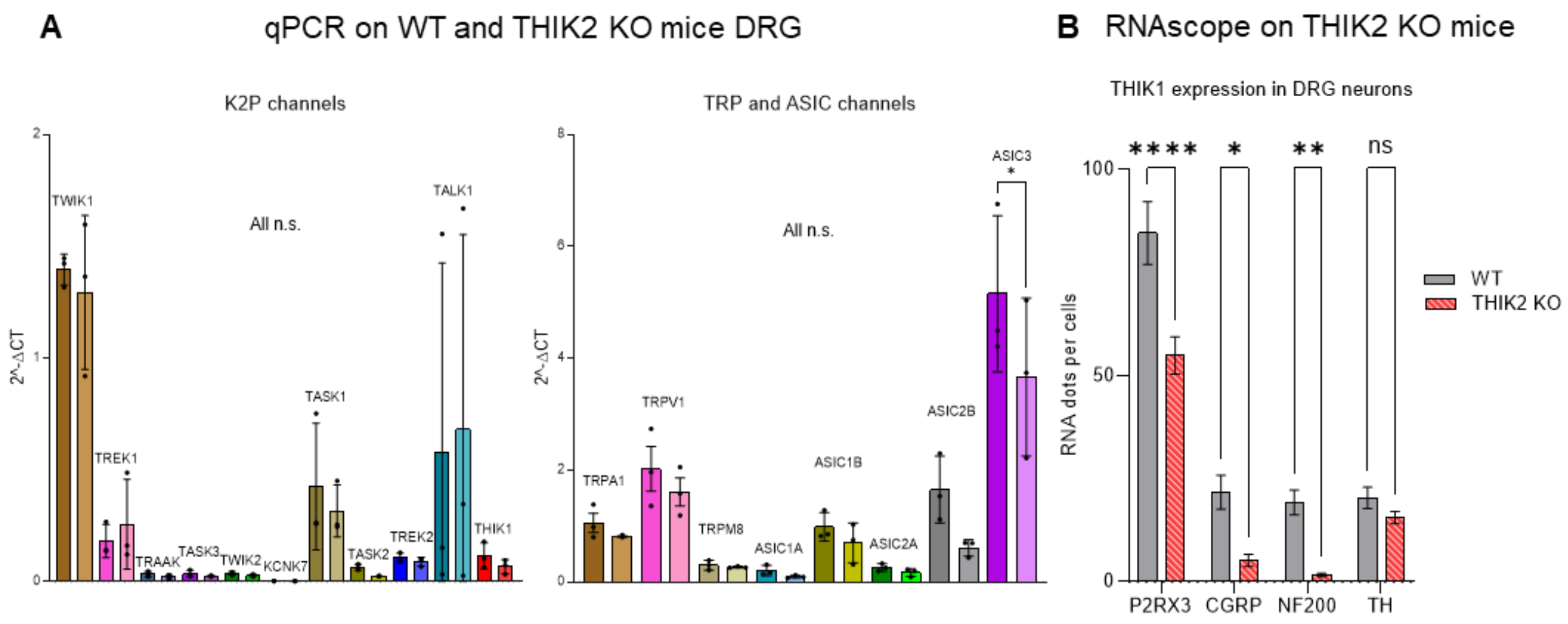

Figure S1. Ion channels mRNA expression levels in DRGs from WT and THIK2$^{-/-}$ mice.

A. Biomark qPCR on WT (n = 3) and THIK2$^{-/-}$ (n = 3) mice DRG showing expression levels of K2P, ASIC and TRP channels mRNA in total DRGs. The results are represented in 2-ΔCT and are normalized by the expression of the TOP1 reporter gene. Mean ± SEM, one-way ANOVA with Sidak's multiple comparisons test, *p<0.05. **p<0.01.

B. Quantification of THIK1-encoding mRNA spots by RNAscope analysis from 3 WT mice DRG and 3 THIK2 KO mice DRG. Mean ± SEM, two- way ANOVA with with Sidak's multiple comparisons test, *p<0.05, **p<0.01 and ****p<0.001. n represent the number of counted neurons; for P2RX3 n=76 WT, n=80 THIK2$^{-/-}$; for CGRP n=71 WT, n=71 THIK2$^{-/-}$; for NF200 n= 80 WT, n=86 THIK2$^{-/-}$; for TH n=48 WT and n=74 THIK2$^{-/-}$.

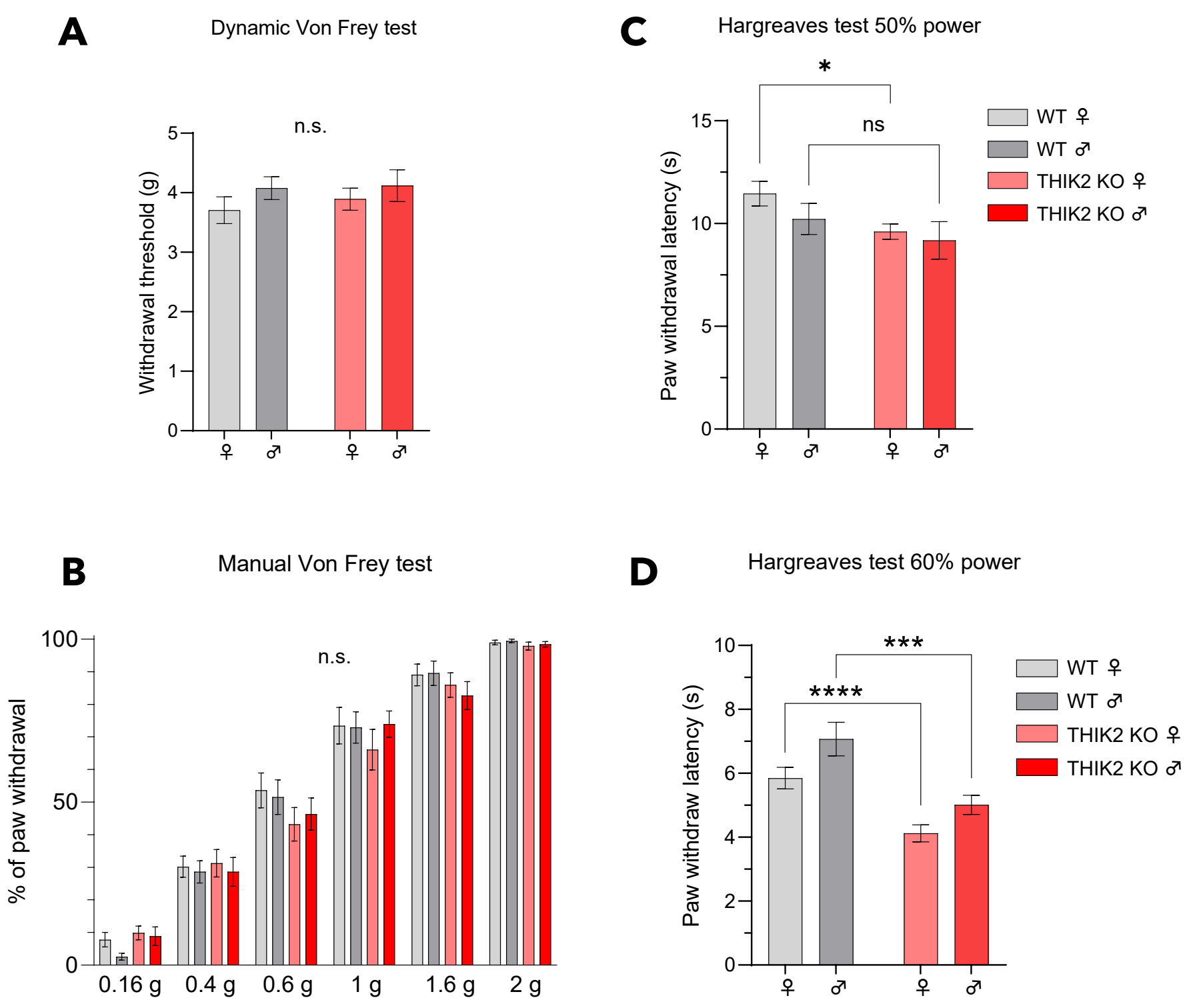

Figure S2. Gender distinction for acute mechanical and thermal sensitivity tests on WT and THIK2$^{-/-}$ mice.

A-B. Paw withdrawal threshold of male and female THIK2$^{-/-}$ mice compared to male and female WT mice in response to mechanical stimuli in dynamic von Frey test (1-7.5 g) (A) or manual Von Frey test (0.16-2 g) (B). Mean ± SEM, Unpaired T-test and two-way ANOVA.

C-D. Paw withdrawal latency of THIK2$^{-/-}$ mice compared to WT mice in response to thermal stimuli, 50% (C) or 60% (D) intensity in Hargreaves test. Mean ± SEM, Multiple T-test, *$p<0.05$, ***$p<0.005$ and ****$p<0.001$. The different sample sizes (n) are shown on graphs.

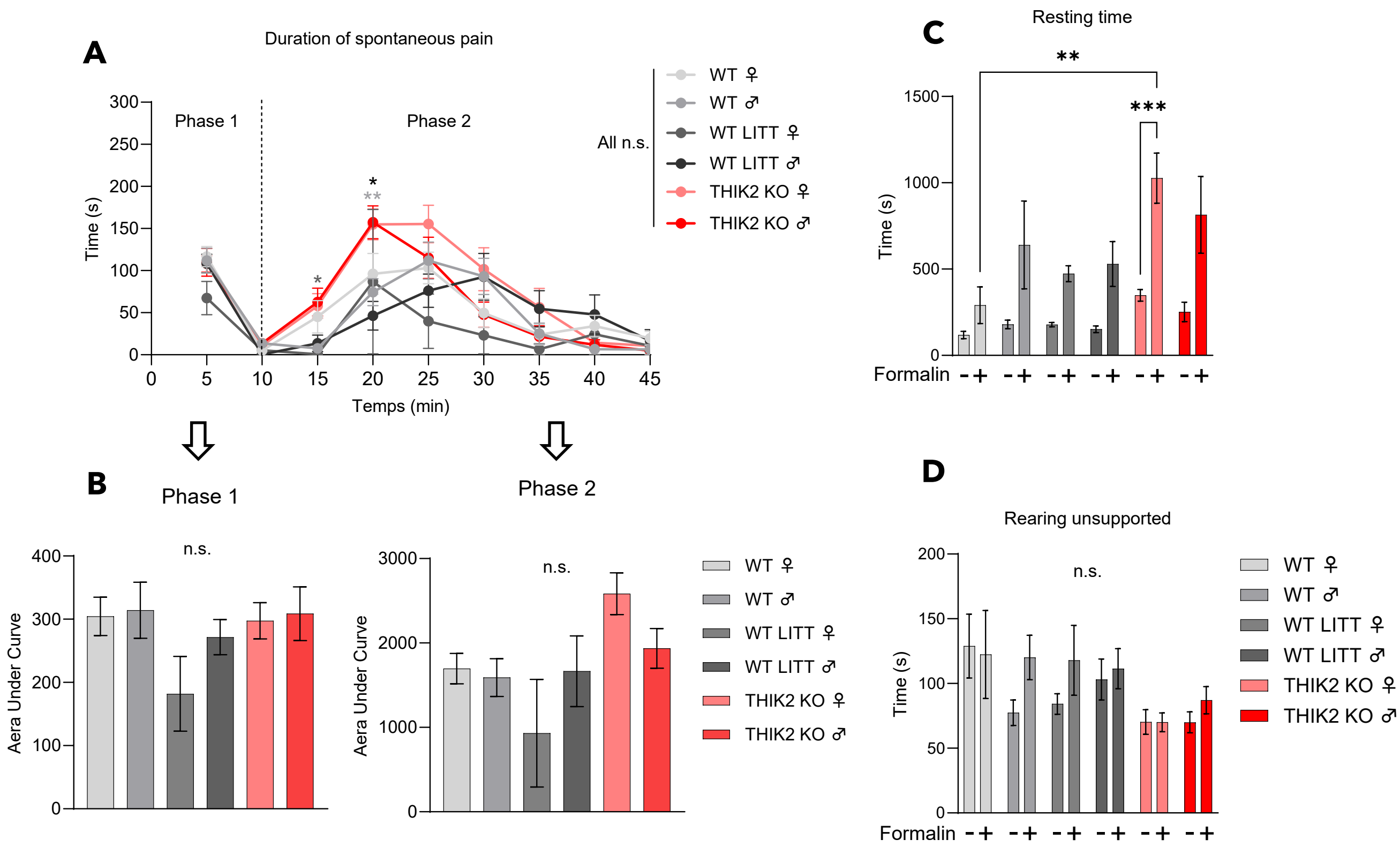

Figure S3. Gender distinction for acute inflammatory spontaneous pain tested on WT, WT littermates and THIK2$^{-/-}$ mice.

A. The pain behaviors measured in the formalin spontaneous pain test were licking, biting, shaking and paw lifting and lasted for 45 min after formalin injection into the left hind paw. Mean ± SEM, two-way ANOVA with Tukey's multiple comparisons test, *p<0.05. **p<0.01.

B. Area under curve from behaviors in acute phase (0-10 min) and inflammatory phase (10-45 min). Mean ± SEM, Multiple T-test.

C-D. Post-formalin recovery was tested by placing the mice in a Phenotyper® observation cage 15 min after the end of the formalin test, resting/prostration time and time spent rearing were measured. Mean ± SEM, two-way ANOVA with Tukey's multiple comparisons test, **p<0.01 and ***p<0.005. The different sample sizes (n) are shown on graphs. For all graphs, WT are wild-type mice from Janvier Labs, and WT littermates are wild-type mice generated from the same lineage as the THIK2$^{-/-}$ mice.

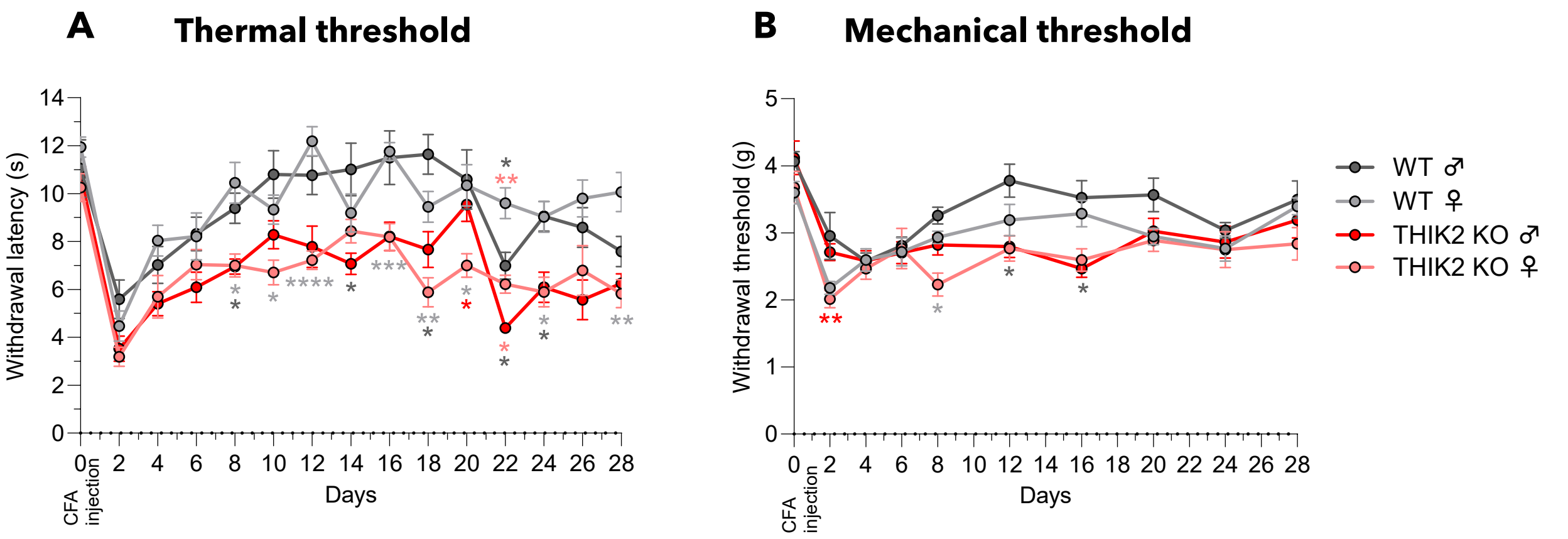

Figure S4: Gender distinction for the chronic inflammatory pain tested on WT and THIK2$^{-/-}$ mice.

A-B. Evolution of paw withdrawal latency during 28 days of male and female WT and THIK2$^{-/-}$ mice in response to thermal stimuli (50 % power) in Hargreaves test (A) or mechanical stimuli (1 g- 7.5 g) in dynamic von Frey test (B) before and after Complete Freund's Adjuvant (CFA) paw injection; *means compared to other genotypes, †means compared to J0. Mean ± SEM, Two-way ANOVA with Bonferroni's multiple comparison test, *p<0.05, **p<0.01, ***p<0.005 and ****p<0.001 and two-way ANOVA with Dunett's multiple comparison test to J0, †p<0.05, ††p<0.01, †††p<0.005 and ††††p<0 .001. Sample size n=10 males WT, n=10 females WT, n=10 males THIK2$^{-/-}$, n=9 females THIK2$^{-/-}$.

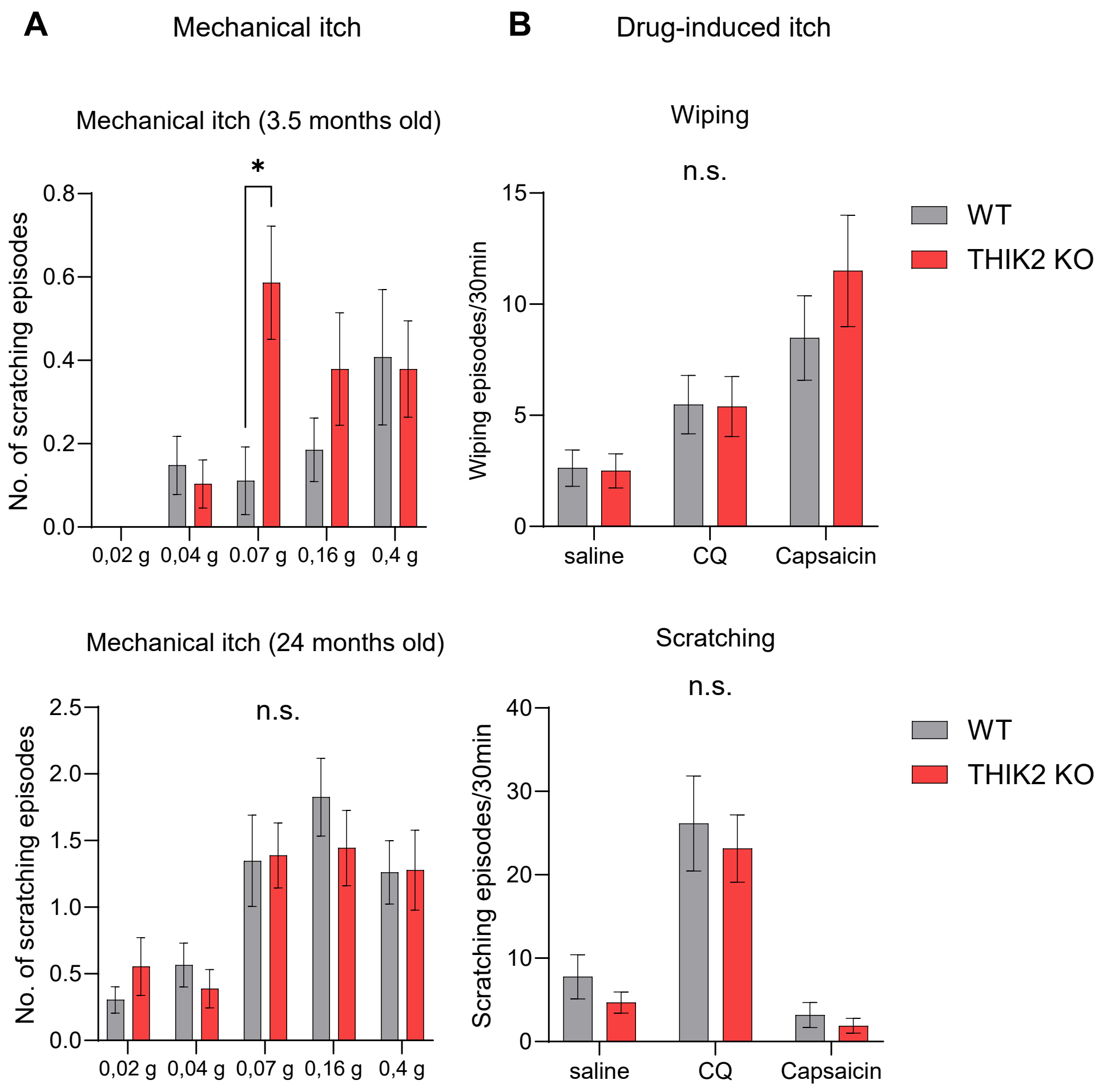

Figure S5. Mechanical and drug-induced itch in WT and THIK2$^{-/-}$ mice.

A. Number of scratching episodes after mechanical itch induced by Von Frey hairs (0.02-0.4 g) at 3.5 months and 24 months. Mean ± SEM, Multiple paired t-test, *<0.05. At 3.5 months old n=27 WT and n=29 THIK2$^{-/-}$ mice; at 24 months old n=23 WT and n=18 THIK2$^{-/-}$ mice.

B. Number of wiping and scratching episodes after saline, chloroquine (CQ) and capsaicin injection. Mean ± SEM, Multiple paired t-test. Sample size n=27 WT and n=28 THIK2$^{-/-}$ mice.

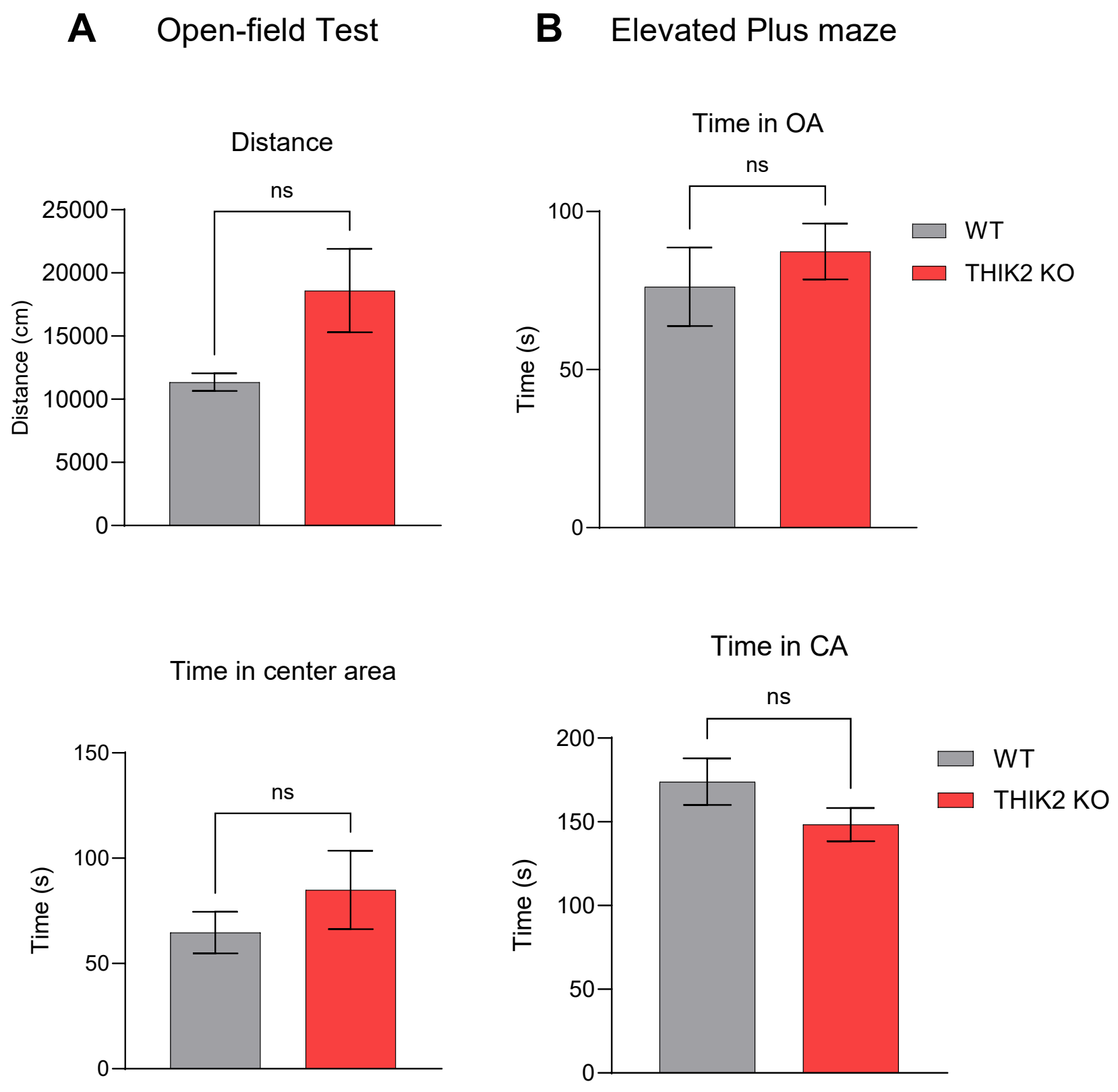

Figure S6. Anxiety behavior and locomotor activity of WT and THIK2$^{-/-}$ Mice.

A. Distance and time spent in center area by THIK2$^{-/-}$ (n = 20) and WT mice (n = 16) in open field test. Mean ± SEM, Unpaired t-test.

B. Time spent in open arms (OA) and closed arms (CA) by THIK2$^{-/-}$ (n = 20) and WT mice (n = 12). Mean ± SEM, Unpaired t-test.

Parameters	WT	THIK2 KO	T-test	Mann-Whitney test
WCC, pF	39.9 ± 0.6	41.6 ± 0.5		*p* = 0.648
R_{input}, M	7.0 ± 0,1	7.1 ± 0.1		*p* = 0.495
Rheobase, pA	70.0 ± 1,8	77.7 ± 2.3		*p* = 0.476
RMP, mV	-50.1 ± 0.3	-48.4 ± 0.4	*p* = 0.503	
APP, mV	31.2 ± 0,6	38.7 ± 0.6		*p* = 0.156
$AP_{threshold}$, mV	-15.5 ± 0.2	-18.5 ± 0.2	*p* = 0.077	
APD_{100}, ms	183.3 ± 2.6	205.4 ± 3.4		*p* = 0.329
Firing frequency, Hz	6.0± 0.2	8 ± 0.2*		***p*** = 0.047
APA, mV	79.0 ± 0.9	87.7 ± 0.9	*p* = 0.186	
AHP amplitude, mV	-11.6 ± 0.2	-12.0 ± 0.2	*p* = 0.804	

Supplementary table. Summary table of WT and THIK2$^{-/-}$ neurons parameters characterized by patch- clamp.

The data are expressed as means ± SEM. WCC, whole cell capacitance; R input, input resistance; Rheobase, minimum current to elicit an action potential; RMP, resting membrane potential; APP, action potential peak; AP threshold, action potential firing threshold; APD100, action potential duration time period from the onset to the point where the repolarization was complete; Firing Frequency, number of AP generated divided by time; APA, action potential amplitude; AHP, afterhyperpolarization. Parameters were determined on non peptidergic IB4-positive WT (n=26) or THIK2$^{-/-}$(n=22) neurons after patch-clamp recordings. Unpaired T-test and Mann-Whitney test, *p<0.05.

Methods

Animal strain and housing

In agreement with national and European Union recommendations 2010/63/EU, experiments have been authorized by "MINISTERE DE L'ENSEIGNEMENT SUPERIEUR ET DE LA RECHERCHE" under the reference number: APAFIS #37438 and APAFIS #44595, after favorable advice of the local Animal Care Committee for animal experimentation. Experiments were performed on WT (WT) C57BL/6J mice (Janvier labs, France) and THIK2 knockout mice (THIK2$^{-/-}$). The THIK2$^{-/-}$βGal$^{+/+}$ mice were generated in Centre d'Immunophénomique (CIPHE) in Marseille. The Kcnk12 gene was inactivated by insertion of a lacZ reporter cassette at the translation start site, combined with premature transcriptional termination via STOP codons and a synthetic polyA signal, thereby abolishing THIK2 expression while preserving endogenous regulatory control. This strategy abolishes normal Kcnk12 expression, generating a reporter-null allele: the gene is functionally inactive, while LacZ reflects endogenous expression. Genotyping is performed by PCR, distinguishing the WT allele (239 bp) from the mutant allele (330 bp). We observed no compensatory upregulation of genes for other neuronal K2P channels in DRGs of THIK2$^{-/-}$βGal$^{+/+}$ mice, particularly THIK1, that is the closest in structure to THIK2 (Supplementary Fig. S1). Males and Females C57Bl/6j mice were housed in groups of 4 or 5 under standard conditions (20-22°C, 40% humidity), with 12h light/dark cycle (lights off at 8 P.M.), with free access to food and water. The mice were separated into 3 groups, THIK2$^{-/-}$, WT mice purchased from Janvier labs and WT littermate mice. They were all tested between 8 and 12 weeks old. Efforts were made to minimize the stress and potential suffering of mice used in this study.

X-Gal DRG staining

To obtain adult tissues, five male THIK2$^{-/-}$βGa$^{l+/+}$ mice were anesthetized with isoflurane (Vetflurane®) and decapitated. Then L1 to L6 DRGs were dissected and were placed in a solution containing X-Gal, which reacts with the enzyme β-galactosidase. After one day, images of whole DRG were taken using a macroscope. DRG were finally placed in cryoprotective solution (OCT) before being frozen and stored at -80°C. DRG were sectioned at 10 µm using a standard cryostat (Leica). Brightfield Images of the slices were taken on a 20X air objective on histology DMD108 Leica microscope. Lumbar DRG from THIK2$^{-/-}$βGal$^{+/+}$ mice were counted in five independent animals. All cell counts were conducted by an individual on OMERO software.

Microfluidic quantitative PCR (Biomark-HD™)

Dorsal root ganglia (DRG) were rapidly dissected and snap-frozen in liquid nitrogen. For RNA extraction, samples were thawed on ice in RNA later. Lysis buffer was freshly prepared under a fume hood by mixing RLT buffer (Qiagen) with 1% β-mercaptoethanol, and 500 µL was distributed into ceramic bead-filled Precellys tubes. DRG tissues were transferred into the lysis tubes using sterilized forceps. Homogenization was performed using a Precellys homogenizer (CORTEX program: 2 × 35 s). The resulting lysates were centrifuged briefly, and 400 µL of the supernatant was collected and mixed with 500 µL of 70% ethanol. The mixture was loaded onto RNeasy Mini spin columns (Qiagen) followed by multiple steps of centrifugations and finally eluted in 30 µL of RNase-free water. cDNAs were obtained from purified RNAs (50 ng) extracted from DRG samples reverse transcribed using the Reverse Transcription kit following the manufacturer's instructions (Qiagen). Thermal cycling conditions were: 42˚C for 15 min, 95˚C for 5 min. cDNAs were preamplified 15 times using the Preamp Master Mix (Fluidigm, PN 100– 5744) according to the manufacturer's protocol. 1 µL of Preamp Master Mix was

combined with 0.5 µL of Pooled Delta gene assay mix and 1.25 µL of cDNA in a 5 µL total volume reaction containing Forward and Reverse primers at 500 nM in a final volume of 200 µL. Thermal cycling conditions were: 95˚C for 2 min followed by 15 cycles of 95˚C for 15 s, 60˚C for 4 min. After each preamplification, reactions were cleaned up from primers through digestion with 2 µL of Exonuclease I (4U/µL) for 30 min at 37°C. Exo I was inactivated for 15 min at 80°C. Samples were diluted 1:20 by adding nuclease-free water up to 100 µL. Real time qPCR using the BiomarkTM HD system was performed using 96.96 IFC (Integrated Fluidic Circuits) following Delta Gene Assays protocol using assays mixes and samples pre- mixes prepared separately. The assays mixes were prepared by mixing 2.5 µL of combined Primer (Forward/Reverse 10 µM) and 2.5 µl 2X Assay Loading Reagent (Fluidigm PN 100– 7611) to a final volume of 5 µL (per reaction). The samples pre-mixes were prepared by mixing 2.5 µL of 2X Ssofast EvaGreen Supermix (BioRad PN 172-5211), 0.25 µL 20X DNA Binding Dye (Fluidigm PN 100-7609) and 2.25 µl of diluted preamplified cDNA to a final volume of 5 µL. After priming the 96.96 IFC, pipet 5 µL of each assay mix and 5 µL of each sample pre- mix into the Assay and Sample inlets of the IFC, respectively. The 96.96 IFC was loaded into the BiomarkTM IFC controller RX and transferred to the BiomarkTM HD apparatus. Thermal cycling conditions were as follows: 50˚C for 120 s, 95˚C for 600 s followed by 20 cycles of 95˚C for 15 s, 60˚C for 1 min.

Sequences primers used (For=forward or sense 5’ to 3’ and Rev=Reverse or antisense, 3’ to 5’)

TWIK1	For=GAGCTGTACAAGATCGGAATCA; Rev=GTCTCCAGGACAACCAGCAT
TREK1	For=AGCATGCCTGCGTCAACT; Rev=TGCGTTTATTGCTGCCACTA
TASK1	For=CCTTCTACTTCGCCATCACC; Rev=GAACATGCAGAACACCTTGC
TRAAK	For=GGTGGGGATCCCACTGTT; Rev=TGGCACATGCCACTTCAA
TASK2	For=CAATGATTTTTGCTGCCACA; Rev=TCTTGGGAGCCACATTGC
TWIK2	For=CCATTGGCCTAGGGGACTAC; Rev=GGCCGAGGAAGAGGTACG
KCNK7	For=CTGTTCACTGCCAGCATCC; Rev=AGAAGGCCTTTCCACCTGA
TASK3	For=ACATCAGCTCCGATGACTACC; Rev=CAGGTGCAGCATGTCCATA

TREK2	For=CCTACTTTGCAGCTGTCCTCA; Rev=GGCTTTGATCTCACCAACCT
THIK2	For=ACCGTGGTGTCGACCATAG; Rev=AACAGTCCGTAGGCGATGAG
THIK1	For=TGAACCTGTTCCACAGAGCA; Rev=AGTGCGAAGAGGCAGATCA
TALK1	For=ATTGGACAGGTGGGAGGAC; Rev=GTCCCCAAGGTCAGGAACA
TRESK	For=TGTGCAACATCCTGAAATGTAAC; Rev=TGTTGCAGATGCTCACACAA
TRPV1	For=GAGTGTGCCTGCACCTAGC; Rev=CTCAGATTCATCCGAGTCTAAGC
TRPA1	For=CGGCCTGAGTTTATGCAGAT; Rev=TGTGCATCCGTCATTGTCTT
ASIC1a	For=GAACTGAAGACCGAGGAGGAG; Rev=GCCGCTCATAGGAGAAGATGT
ASIC1b	For=TCAGCTACCCTGACTTGCTCTA; Rev=GAGCGGTTGTAGAAACGATGGA
ASIC2a	For=CGATGGACCTCAAGGAGAGC; Rev=ATACACGAAGATGTGGCGGAT
ASIC2b	For=CTTGCTGTTGTCCTGGTCCT; Rev=TTGTTGTTGCACACGGTGAC
ASIC3	For=TTCACCTGTCTTGGCTCCTC; Rev=TGACTGGGGATGGGATTTCTAAG
TOP1	For=GCCTCCATCACACTACAGCA; Rev=TTCGCTGGTACATTCTCATC

Open field test

Open field experiments were done to assess the locomotor activity and anxiety behavior. Mice were placed in open field boxes (45 x 45cm) with illumination and video tracking from above. Locomotion activity of mice was analyzed with Noldus® Ethovision XT Software. Total traveled distance and center entry in 1h were scored.

Elevated Plus maze

Elevated Plus maze were done to measure anxiety behavior. Mice were placed in a plus-shaped apparatus elevated 50 cm above the ground with two open arms (30 x 5 cm) and two closed arms (30 x 5 cm) with 20 cm walls with illumination and video tracking from above. Time spent into closed and open arms was recorded for 5 min and analyzed with Noldus® Ethovision XT Software.

Mechanical itch

Mechanical itch was assessed to test alloknesis [1], the itch sensation evoked by innocuous mechanical stimulation such as light touch. Mice were shaved 24 hours before experiment. On the testing day, mice were placed in small chamber with bedding and Von Frey hairs were

applied (0.02-0.4 g) to induce itch response. Mice received 3 stimulations for each von Frey filament. Number of scratch episodes were scored in response to innocuous mechanical stimulation.

Drug-induced pain/itch response

Cheek skin assay was used to assess pain or itch responses after drug injection as described by [2]. Mice were shaved 24 hours before experiment. Mice received an intradermal injection of saline (NaCl 0.9%,10 µL), capsaicin (50 µg/µL) or chloroquine (200 µg/µL) to their cheek skin. After drug injection, numbers of grooming, wiping or scratching were recorded for 30 min and analyzed.

References

[1] Akiyama T, Carstens MI, Ikoma A, Cevikbas F, Carstens E. Mouse model of touch-evoked itch (alloknesis). 2013.

[2] Lin S-H, Steinhoff M, Ikoma A, Chang Y-C, Cheng Y-R, Kopparaju RC, Ishii S, Sun W-H, Chen C-C. Involvement of TRPV1 and TDAG8 in Pruriception Associated with Noxious Acidosis. 2017.
